# Coupling analysis of heart rate variability and cortical arousal using a deep learning algorithm

**DOI:** 10.1371/journal.pone.0284167

**Published:** 2023-04-06

**Authors:** Jiayan Huo, Stuart F. Quan, Janet Roveda, Ao Li

**Affiliations:** 1 Biomedical Engineering, The University of Arizona, Tucson, AZ, United States of America; 2 Division of Sleep and Circadian Disorders, Departments of Medicine and Neurology, Brigham and Women’s Hospital, Harvard Medical School, Boston, MA, United States of America; 3 Asthma and Airway Disease Research Center, College of Medicine, The University of Arizona, Tucson, AZ, United States of America; 4 Electrical and Computer Engineering, The University of Arizona, Tucson, AZ, United States of America; 5 BIO5 Institute, The University of Arizona, Tucson, AZ, United States of America; Charite Medical University Berlin, GERMANY

## Abstract

Frequent cortical arousal is associated with cardiovascular dysfunction among people with sleep-disordered breathing. Changes in heart rate variability (HRV) can represent pathological conditions associated with autonomic nervous system dysfunction. Previous studies showed changes in cardiac activity due to cortical arousals. However, few studies have examined the instantaneous association between cortical arousal and HRV in an ethnically diverse population. In this study, we included 1,069 subjects’ full night ECG signals from unattended polysomnography in the Multi-Ethnic Study of Atherosclerosis dataset. An automated deep learning tool was employed to annotate arousal events from ECG signals. The etiology (e.g., respiratory, or spontaneous) of each arousal event was classified through a temporal analysis. Time domain HRVs and mean heart rate were calculated on pre-, intra-, and post-arousal segments of a 25-s period for each arousal event. We observed that heart rate and HRVs increased during the arousal onsets in the intra-arousal segments, regardless of arousal etiology. Furthermore, HRVs response to cortical arousal occurrence differed according to gender and the sleep stages in which arousal occurred. The more intense HRVs variation due to arousal in females can contribute to a potentially stronger association between arousal burden and long-term mortality. The excessive abrupt sympathetic tone elevation in REM caused by arousal may provide insights on the association between sleep and sudden cardiac death.

## Introduction

Cortical arousals during sleep are transient events that indicate temporary intrusion of wakefulness [1]. Sudden cortical arousals can be spontaneous or triggered by sleep-disordered breathing (SDB) [2], periodic leg movements (PLM) [3], bruxism [4], pain [5], and noise [6]. Frequent cortical arousals can cause sleep fragmentation, poor sleep quality, and insufficient sleep. Additionally, arousals occurring in obstructive sleep apnea (OSA) patients are associated with cardiovascular dysfunction [7].

Cortical arousals are currently characterized by electroencephalography (EEG) in polysomnography (PSG) or level 2 home sleep tests (HST) [8]. Several studies have found cortical arousals can impact cardiac activity [9–12], therefore cortical arousal events related to autonomic nervous system (ANS) activity can be detected via cardiac activity variations. Heart rate variability (HRV) represents the beat-to-beat fluctuation of RR intervals and can reflect ANS activity [13–15]. However, there are few data from racially/ethnically diverse and/or multi-center cohorts, and the instantaneous association between cortical arousal and heart rate and time domain HRV in the general population is unknown.

An emerging trend in healthcare is employment of deep learning techniques on large amounts of clinical data to extract interpretable information and discover new knowledge [16]. The deep learning approach is a data-driven algorithm that does not need domain knowledge and can automatically learn from raw biomedical signals. This approach employs a deep neural network consisting of multiple layers. Each layer includes multiple filters designed to extract features at different levels. For example, in a classification task, higher-level layers amplify aspects of the crucial inputs for discrimination and suppress irrelevant variations. Compared to human-designed filters, a deep neural network discovers intricate patterns in large data sets using backpropagation algorithms to indicate how a network should change its filter weights [17]. Currently, however, most studies using a deep learning approach in the sleep field only focus on identifying individuals with sleep disorders. Exploring novel uses of deep learning in analyses of signals recorded from sleep studies is an emerging field with the potential to provide insight into the pathophysiology of sleep disorders.

The objective of this study was to investigate the capability of applying a deep learning algorithm to electrocardiographic signals (ECG) in order to better understand the coupling between cortical arousal and HRV in the general population. Because cortical arousals are transient events in sleep, the duration of cardiac changes is dynamic and short. Therefore, we designed a deep learning algorithm to accurately locate the region of interest on the ECG for the coupling analysis. Then, we performed an HRV analysis on the selected ECG segments. Because cortical arousals can be elicited by respiratory disturbances or non-respiratory disturbances, we firstly compared the difference in cardiac responses between these two conditions. Subsequently, we stratified the general population into subpopulations based on the gender to compare differences in their cardiac responses. Because studies have found that the sleep stages can also affect HRV [18, 19]. we also compared the cortical arousal induced cardiac responses between rapid eye movement (REM) and non-rapid eye movement (NREM) sleep.

## Methods

### Data source

We used the Multi-Ethnic Study of Atherosclerosis (MESA) dataset for deep learning model development, testing, and statistical analyses. MESA is a multi-center collaborative longitudinal study that evaluates the progression of subclinical to clinical cardiovascular disease [20]. Between 2010 to 2012, 2237 of the original 6814 participants which included black, white, Hispanic, and Chinese-American men and women were enrolled in the Sleep Exam substudy. The Sleep Exam included unattended full overnight polysomnography (PSG), 7-day wrist-worn actigraphy, and a sleep questionnaire. The single-lead electrocardiogram (ECG) from the PSG record was used to identify arousal events where ANS activities were affected by cortical arousals. The sampling frequency of ECG was 256 Hz. The PSG and demographic data of participants can be obtained publicly from https://sleepdata.org/datasets/mesa.

Certified scorers visually scored cortical arousals manually based on American Academy of Sleep Medicine (AASM) criteria [21]. The AASM defines cortical arousal as an abrupt shift in EEG frequency, which may include alpha and/or theta waves and/or delta waves and/or frequencies greater than 16 Hz lasting at least 3 seconds and starting after at least 10 continuous seconds of sleep. In rapid-eye-movement (REM) sleep, an increase in the EMG signal is also required. Interscorer reliability for identification of arousals is presented in the S1 File.

### Subject exclusion

**Fig 1:**
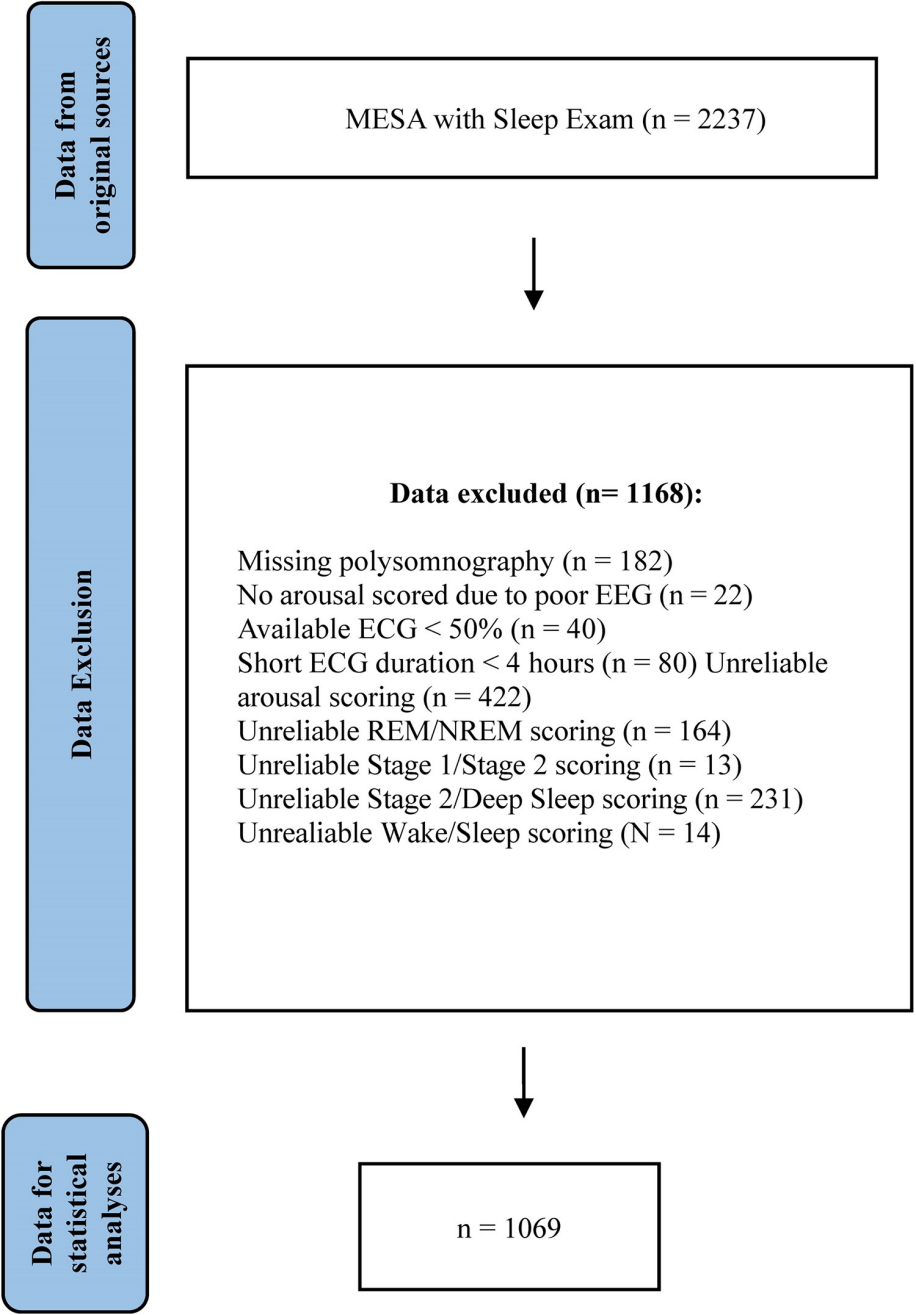
Flow chart of data inclusion in this study. ECG: electrocardiogram; EEG: electroencephalogram.

We excluded 1,168 subjects due to unreliable and/or missing scored events. The details are provided in Fig 1. The final dataset consisted of 1,069 participants for cortical arousal and HRV coupling analyses. The subject’s exclusion seems to be random regarding to gender or ethnicity. The demographic statistical of subjects before and after the exclusion process was included in the S1 Table in S1 File.

### Model architecture

We used the pre-trained deep learning model reported in our previous study for arousal detection [22]. Specifically, the model took a 256 Hz ECG signal as input and output the sequence of arousal probabilities at a one-second resolution based on the presence or absence of an arousal previously scored in the MESA dataset. The detailed model architecture is described in S1 Fig in S1 File.

### Statistical analyses

**Fig 2:**
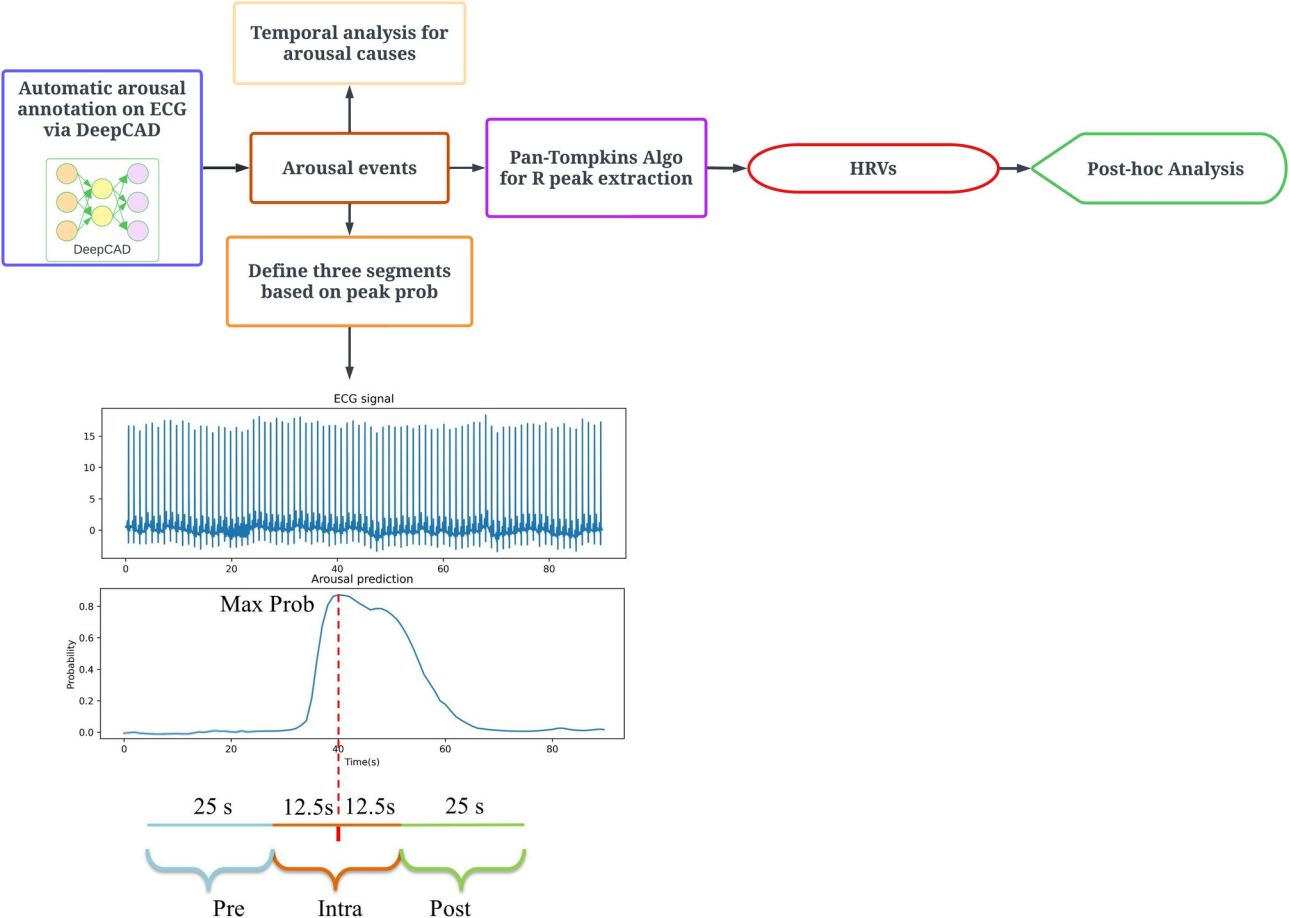
Data processing flowchart for statistical analyses. Algo: Algorithm; HRV: Heart Rate Variability; Prob: probability.

Fig 2 shows the procedures for data processing and statistical analyses. The output of the deep learning model was a sequence of arousal probability scores between 0 and 1. A decision threshold was chosen using the geometric mean of probability scores to achieve a balance between sensitivity and specificity, calculated by the maximum values of true positive rate * (1 –false-positive rate). Any score beyond the cut-off threshold indicated the presence of arousal at the corresponding second. Moreover, we excluded false positive arousal predictions of the model. These false positives may be linked with the cardiac activity changes due to other events, such as sleep stage transition [14]. They hence cannot appropriately reflect the association between HRV and cortical arousals.

We compared the HRV variations before and after the arousal events which were automatically annotated by the deep learning model. All annotated arousal events met the criteria that the duration was > 3 seconds and the interval between two arousal events was > 11 seconds on ECG [23]. For each arousal event, three consecutive segments of equal length, pre-, intra-, and post-arousal, were defined based on the maximum predicted probability of arousal via the deep learning model, as shown in Fig 2. The impact of cortical arousals on HRV was examined by comparing HRV extracted from these three ECG segments. Based on the previous studies on ultra-short term HRV [12, 24], a segment of 25 seconds is sufficient for HRV time domain analysis. Additionally, to reveal the association between different types of arousals and HRV, a temporal analysis was conducted to determine the factor that resulted in each arousal. The etiology for arousal was searched 10 seconds prior to the arousal onset as the autonomic nervous system may have a delayed response to cortical arousal [25]. The etiologies were determined using a priority order of OSA, central sleep apnea (CSA), hypopnea, PLM, undefined oxygen desaturation (UOD), and spontaneous. Given that apneas and hypopneas were scored with at least a 3% oxygen desaturation in MESA dataset, UOD arousals may also be triggered by events such as OSA, CSA, or hypopnea but with an oxygen desaturation < 3%. Therefore, a secondary search for UOD arousal was necessary to possibly identify the origin of an UOD arousal. Specifically, we further searched another 10 seconds prior to the oxygen desaturation associated with UOD arousals. The UOD arousal events were re-classified if any events happened in the 10-second searching window, such as OSA or CSA.

After arousal events were identified and classified, the Pan-Tomkins algorithm [26] was implemented using python to extract R peaks from pre-, intra-, and post-arousal segments for HRV analyses. Data exclusion is essential for HRV analysis as abnormal beats may compromise the reliability of HRV [27]. Karavirta stated that RR intervals between 400 to 2000 ms can be considered normal at rest [28]. Therefore, the arousal events were excluded if abnormal RR intervals (<400 ms or >2000 ms) were found in either of the three segments. Table 1 shows the descriptions and expressions of HRV features and heart rate involved in this study.

**Table 1:** The time domain parameters in this study.

*Parameters*	*Description*	*Expression*	*Unit*
*SDNN*	Standard deviation of normal RR intervals	$\sqrt{\frac{1}{N-1}\sum_{i=1}^{N}{\left(RR_{i}-\bar{RR}\right)}^{2}}$	ms
*RMSSD*	Square root of the mean squared differences between successive RR intervals	$\sqrt{\frac{1}{N-1}\sum_{i=1}^{N-1}{\left(RR_{i}-RR_{i+1}\right)}^{2}}$	ms
*pNN50*	Number of successive RR intervals pairs differing more than 50 ms divided by the total number of RR intervals	$Count(|RR_{i+1}-RR_{i}|>50\;\mathrm{ms})/(N-1)$	%
*HR*	Heart rate	$60*1000/\bar{RR}$	beats per minute

N: total normal R peaks within the ECG segment; $\bar{RR}$: average of RR intervals.

We first confirmed the data normality using Sharpiro-Wilk test [29]. Then we applied paired t-tests to compare the means of HRV parameters between pre-/intra-, and intra-/post-arousal segments of the same type of arousal. T-tests were used for the comparisons of means of HRVs between different genders and sleep stages. We also implemented an alpha adjustment based on Bonferroni corrections as multiple comparisons (192 in total) were made in this study. Therefore, the p-values were adjusted with a multiplier of 200. We considered that adjusted p-value < 0.05 indicated statistical significance in analysis. Model testing and analyses were performed using Python v3.6 with package PyTorch v1.8, Scikit-learn v0.24, and SciPy v1.4.

## Results

### Subject characteristics

Subjects’ characteristics, including arousal duration extracted from annotation files, are presented in Table 2 Subjects were older; a slight majority of the 1,069 subjects were female and had at least moderate sleep apnea (apnea hypopnea index [AHI] ≥ 15 /h)

**Table 2:** Descriptive characteristics of the subjects.

*Characteristics*	*Subjects (n = 1069)*
*Female (%)*	53.5%
*AHI > = 15 (with > = 3% oxygen desaturation or with arousal)*	55.9%
*Age (mean ± SD)*	69.0 ± 8.9
*Race*	
*Black, African American*	25.7%
*Caucasian, White*	40.2%
*Chinese American*	11.3%
*Hispanic*	22.8%
*Arousal index (mean ± SD)*	22.2±11.8
*Arousal event duration [seconds] (mean ± SD)*	9.08±5.95
*Number of OSA Arousals*	9745
*Number of CSA Arousals*	851
*Number of Hypopnea Arousals*	21646
*Number of PLM Arousals*	5304
*Number of UOD Arousals*	25825
*Number of Spontaneous Arousals*	22563
*Number of NREM Arousals*	76279
*Number of REM Arousals*	9655
*Number of Arousals for Male*	44889
*Number of Arousals for Female*	41045

AHI: Apnea-Hypopnea Index; OSA: Obstructive Sleep Apnea; CSA: Central Sleep Apnea; PLM: Periodic Leg Movements; REM: Rapid Eye Movement; NREM: Non-rapid Eye Movement; UOD: undefined oxygen desaturation.

### Model performance and arousal inclusion

**Fig 3:**
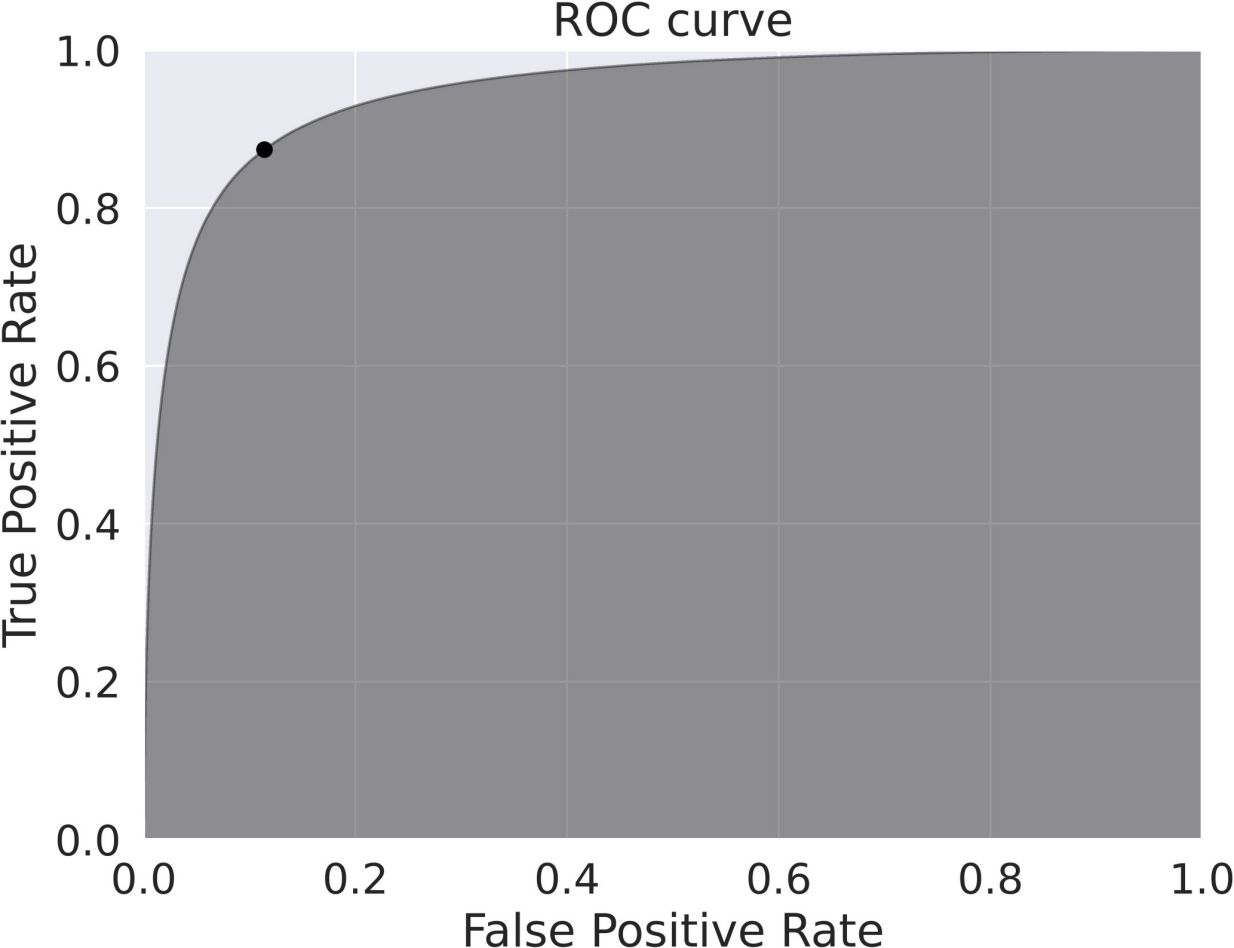
The receiver operating characteristic (ROC) curve for arousal classification using the deep learning model.

Fig 3 shows the receiver operating characteristic (ROC) curve of the deep cortical arousal detection algorithm (DeepCAD) for arousal classification; the optimal cut-off threshold (0.063) is depicted as the black dot. With the selected threshold, we initially included 85.2% (106,971 of 125,524) of the arousal events. We further excluded 16.76% (21,037 of 125,524) arousal events due to the abnormal RR intervals on corresponding ECG segments. Finally, 68.4% (85,934 of 125, 524) of original arousal events were selected for final analysis. The distribution of arousal etiologies and arousal lengths are shown in Table 2 and S2 Fig in S1 File, respectively. Most arousal events lasted less than twenty seconds, implying a 25-second intra-segment is sufficient to record the HRV changes resulting from arousal. According to Table 2, UOD, spontaneous, and hypopneic arousals were the majority of arousal events. Furthermore, greater than 85% of UOD arousals were not related to other arousal etiologies after the secondary search.

### Comparison of HRV parameters among arousals etiologies

The HR and following HRV parameters were analyzed for each arousal: SDNN, RMSSD, pNN50 (See Abbreviations for definitions). Table 3 compares the HRV parameters between two segments pairs, pre- versus intra-, and intra- versus post-arousal, within the specific type of arousal event. The means of pNN50, and HR were significantly higher during the arousal onset compared to both pre- and post-arousal segments in all types of arousals. Except for CSA induced arousals, the mean of SDNN in intra-arousal segment was also significantly higher compared to pre- and post-arousal segments. The mean of RMSSD were observed a significant increase during intra-arousal segment in OSA, UOD and spontaneous arousal events whereas no significant increasing were found in CSA arousal events. No significant differences were found between pre- and post-arousal segments. P-values after adjustment and effect sizes (Cohen’s d) for HRV parameter comparisons within pairwise segments are presented in S2 Table in S1 File. As shown in Table 3, pairwise comparison showed a significant change in the HRVs and heart rate in the presence of all types of arousals, suggesting an elevation of cardiac sympathetic tone due to arousal onset.

**Table 3:** Comparison of HRV parameters and heart rate between pre- vs intra-arousal and intra- vs post-arousal.

	*Pre-arousal*	*Intra-arousal*	*Post-arousal*	*Paired t-test result*
	Mean ± Standard deviation			
*OSA*				
SDNN [ms]	56.77 ± 50.60	74.87 ± 45.69	55.36 ± 48.19	a, b
RMSSD [ms]	55.31 ± 79.80	58.96 ± 70.62	54.09 ± 77.71	a, b
pNN50 [%]	15.01 ± 20.83	18.19 ± 20.14	14.60 ± 20.81	a, b
HR [bpm]	63.47 ± 8.35	67.73 ± 8.23	63.66 ± 8.50	a, b
*CSA*				
SDNN [ms]	70.08 ± 65.91	73.93 ± 58.97	62.71 ± 60.24	b
RMSSD [ms]	69.25 ± 104.47	72.45 ± 98.00	67.11 ± 98.90	
pNN50 [%]	14.54 ± 21.42	18.02 ± 22.97	14.60 ± 21.88	a, b
HR [bpm]	63.79 ± 8.87	66.59 ± 8.40	63.49 ± 8.86	a, b
*Hypopnea*				
SDNN [ms]	45.90 ± 47.16	58.76 ± 44.99	48.88 ± 47.38	a, b
RMSSD [ms]	48.54 ± 75.30	50.36 ± 70.74	49.11 ± 75.66	a
pNN50 [%]	11.92 ± 19.22	12.92 ± 18.36	12.01 ± 19.03	a, b
HR [bpm]	65.82 ± 8.65	68.04 ± 8.68	65.71 ± 8.69	a, b
*PLM*				
SDNN [ms]	50.37 ± 45.60	61.46 ± 42.57	50.60 ± 43.41	a, b
RMSSD [ms]	48.39 ± 69.73	50.55 ± 65.22	47.83 ± 67.87	b
pNN50 [%]	13.10 ± 19.08	14.52 ± 18.97	13.02 ± 19.03	a, b
HR [bpm]	64.62 ± 8.56	65.81 ± 8.51	64.53 ± 8.70	a, b
*UOD*				
SDNN [ms]	52.26 ± 52.67	65.89 ± 48.50	53.19 ± 51.66	a, b
RMSSD [ms]	54.16 ± 84.85	55.89 ± 77.77	54.32 ± 83.75	a, b
pNN50 [%]	13.38 ± 20.49	14.84 ± 19.76	13.39 ± 20.28	a, b
HR [bpm]	64.89 ± 8.68	67.89 ± 8.73	64.81 ± 8.76	a, b
*Spontaneous*				
SDNN [ms]	43.54 ± 46.80	60.43 ± 45.03	48.25 ± 46.47	a, b
RMSSD [ms]	47.88 ± 74.16	50.19 ± 68.98	47.36 ± 71.49	a, b
pNN50 [%]	12.53 ± 20.02	13.64 ± 18.75	12.60 ± 19.55	a, b
HR [bpm]	65.08 ± 8.49	67.22 ± 8.61	65.18 ± 8.74	a, b

Paired t-test was considered statistically significant if adjusted p-value < 0.05

^a^ Statistical significance difference between pre- and intra-arousal event

^b^ Statistical significance difference between intra- and post-arousal event

bpm: Beats per minute; CSA: Central Sleep Apnea; HR: Heart rate; OSA: Obstructive Sleep Apnea; PLM: Periodic Leg Movements; pNN50: Number of successive RR intervals pairs differing more than 50 ms divided by the total number of RR intervals; RMSSD: Square root of the mean squared differences between successive RR intervals; SDNN: Standard deviation of normal RR intervals; UOD: Undefined oxygen desaturation.

### Comparison of HRV parameters associated with different types of arousals between male and female

**Fig 4:**
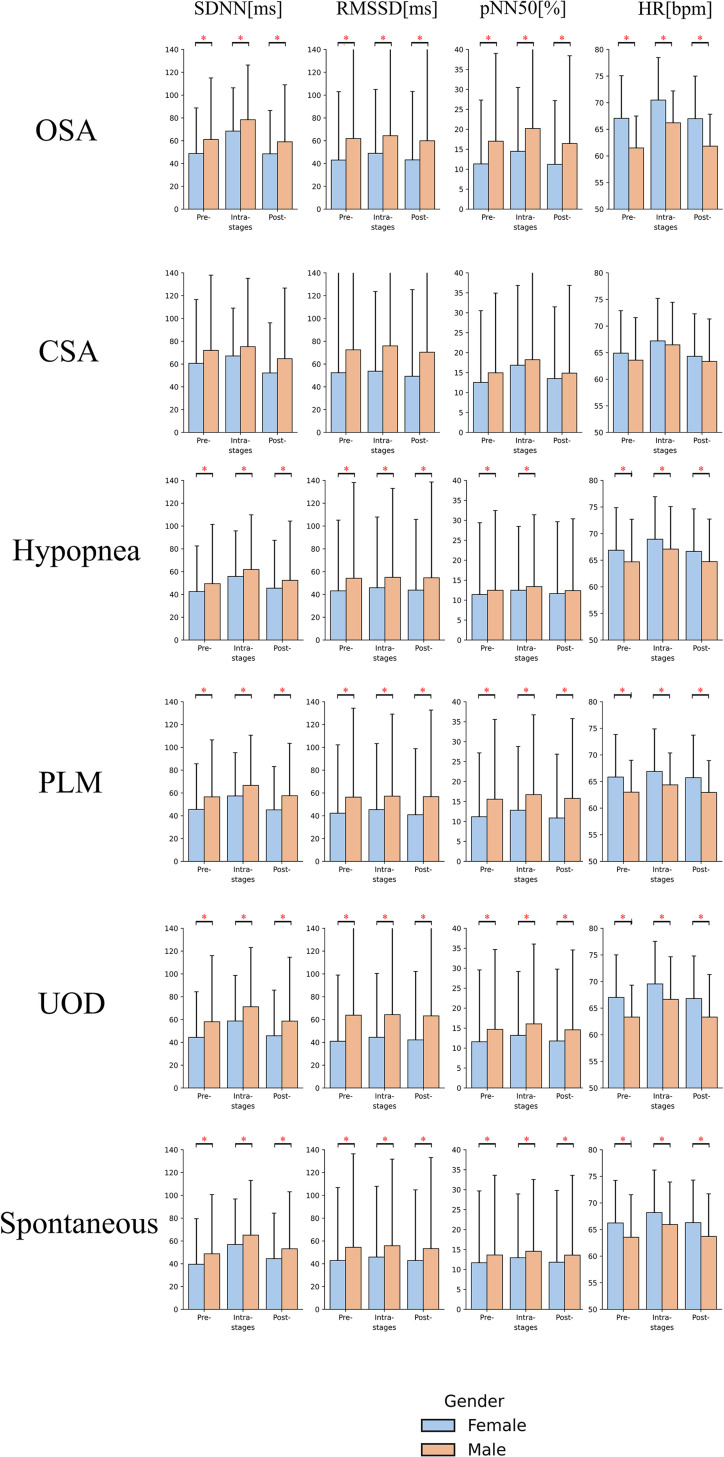
Bar plot of parameters calculated from ECG signal across three defined segments of different arousal events between male and female. The asterisks indicate the pairwise statistical significance (adjusted p-value < 0.05). Error bar indicates standard deviation. CSA: Central Sleep Apnea; PLM: Periodic Leg Movements; SDNN: Standard deviation of normal RR intervals; RMSSD: Square root of the mean squared differences between successive RR intervals; pNN50: Number of successive RR intervals pairs differing more than 50 ms divided by the total number of RR intervals; HR: Heart rate.

Fig 4 shows the comparison of the means of HRVs between genders across three segments, pre-, intra-, and post-arousal. Means and standard deviations of HRVs, and adjusted p-values with effect sizes (Cohen’s d) for HRV parameter comparison within pairwise segments for different genders are shown in S3 and S4 Tables in S1 File, respectively. All means of HRVs and heart rate were greatest during the arousal regardless of gender. Except for CSA arousal events, the means of HRVs, including SDNN, RMSSD and pNN50 were significantly higher in males regardless of arousal segments, whereas the mean of heart rate was higher in the female. Moreover, more intensive variation was observed in females regardless of arousal types. Taking OSA arousal as an example, the mean of SDNN increased by 40.24% [before arousal: 48.77, arousal onset:68.40] in females while it increased by 28.35% [before arousal: 61.08, arousal onset:78.40] in males. The mean of RMSSD among females increased by 13.74% [before arousal: 43.03, arousal onset: 48.94] whereas that among males increased by only 3.93% [before arousal:61.94, arousal onset: 64.38]. A similar result was also observed in the mean of pNN50 where females increased by 27.85% [before arousal: 11.33, arousal onset: 14.48] whereas males increased by 18.74% [before arousal: 17.02, arousal onset: 20.21].

### Comparison of HRV parameters associated with different types of arousals between REM/NREM stages

**Fig 5:**
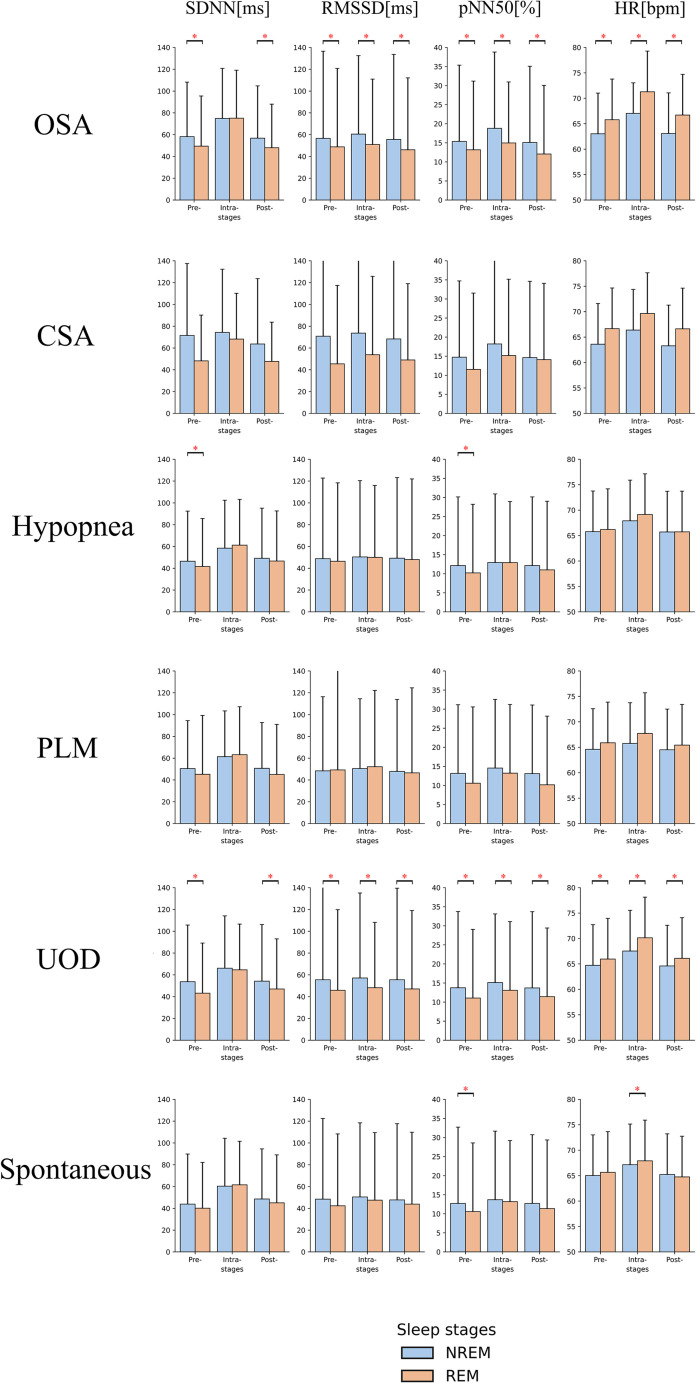
Bar plot of parameters calculated from ECG signal across three defined segments of CSA induced arousal events between REM and NREM. The asterisks indicate the pairwise statistical significance (adjusted p-value < 0.05). Error bar indicates standard deviation. CSA: Central Sleep Apnea; SDNN: Standard deviation of normal RR intervals; RMSSD: Square root of the mean squared differences between successive RR intervals; pNN50: Number of successive RR intervals pairs differing more than 50 ms divided by the total number of RR intervals; HR: Heart rate. REM: Rapid Eye Movement; NREM: Non-rapid Eye Movement.

The comparison of HRV means across the three segments in REM and NREM stages are shown in Fig 5. For all arousal types, the means of SDNN, RMSSD, and pNN50 were lower in REM compared to NREM, while the mean of heart rate was higher in REM. The only exception was for PLM arousal events where the means of RMSSD were nearly the same. Furthermore, at arousal onset, the variations of SDNN were larger in REM than in NREM stage regardless of arousal types. For instance, in OSA-induced arousal events, the mean of SDNN during REM stage increased by51.82% [from 49.46 to 75.09] due to arousal onset while NREM only increased by 28.62% [from 58.17 to 74.82]. No significant differences were observed regarding the mean of SDNN in intra-arousal segments due to more intensive variations of SDNN during REM stage. Means with standard deviation, and adjusted p-values and effect sizes (Cohen’s d) for HRV parameter comparisons within pairwise segments for REM/NREM are presented in S5 and S6 Tables in S1 File.

## Discussion

We demonstrate that the deep learning model can provide insights into the instantaneous association between cortical arousal and HRV using a single-lead ECG. Importantly, comparisons of HRVs between various arousal etiologies and different subpopulations further reveal the magnitude and characteristics of associations between cortical arousal and HRV parameters in the general population.

Previous studies examined the association between arousal and cardiovascular activity change either using short term (~ 5 min) [9] or long-term (overnight) ECG measurements in a general population [11] or ultra-short-term (< 5mins) ECG measurements only among OSA populations [12]. The instantaneous response of cardiovascular activity to cortical arousal among a general population is still unknown. In the present study, we employed a deep learning model, DeepCAD, to annotate arousal events with 1-second resolution on the ECG, making it possible to analyze the short-term effects that arousal onsets have on cardiac activation among the general population. We also distinguished the arousal based on the causes via a temporal analysis.

For the overall population, except for the RMSSD of OSA induced arousals, we found significant variations in HR and HRVs in every type of arousal, with increasing magnitude during arousal onset and reduced to baseline level after arousal. The results of HR change were consistent with a previous observation [9] that arousal increases sympathetic tone. A substantial impact from parasympathetic withdrawal is represented by the rapid surge in heart rate during arousal onset [30]. It is noteworthy that our analysis revealed an increase in heart rate variability (HRV) during intra-arousal segments. Arousal may also lead to the induction of abnormal heartbeat patterns [31, 32], resulting in irregular RR intervals. Given that our analysis of HRVs was based on ultra-short-term recordings, this may have a more significant impact compared to HRV calculations using a longer window size. It should also be noted that the magnitude of variations for CSA-induced arousal are less intense in the SDNN and RMSSD components of HRV in comparison to other arousal types. This is likely to result from clinical differences between different types of arousals [33].

Stratified analysis based on different genders revealed cardiovascular response differences to the occurrences of cortical arousal. The result in Fig 4 is consistent with documentation that females have a higher heart rate but lower HRVs [34, 35]. However, we did not observe the significant differences between male and female on the HRVs and HR among CSA-induced arousals. One of the possible reasons is the limited number of the CSA arousal events, as shown in Table 2. The different intensity of HRV responses to cortical arousal onsets resulted in statistically significant changes among the pre-, intra- and post- segments. Specifically, the SDNN, RMSSD and pNN50 components of females increased more than those of males in all types of arousals, which can potentially result in different levels of long-term cardiac burden. Shahrbabaki et. al [11] calculated arousal burden through the cumulative duration of arousal events and total sleep time, and observed females have a stronger association between arousal burden and long-term cardiovascular morbidity as well as overall mortality in comparison to males. One possible reason is the different sympathetic nervous system responses during arousal between males and females as discussed above.

The result in Fig 5 showed the lower HR and higher HRVs in NREM and opposite trends in REM sleep stage, which is consistent with the sympathetic nervous system activity during REM sleep. Moreover, we observed greater variations of HRVs due to arousal onset during REM compared to NREM sleep in particular with SDNN in all types of arousals. Though the sympathetic activation in REM is well known, the instantaneous and intensive variation of the sympathetic tone due to cortical arousal in REM may further stress cardiac function and potentially be a factor in inducing sudden cardiac death [36]. Thus, it is essential to understand the spontaneous impacts of sleep stages and arousal on HRVs and their potential adverse effects on cardiac function.

Several limitations of this study should be noted. First, the conclusion depends on the reliable arousal and sleep stage scoring as most of the excluded subjects were due to the arousal/sleeping stage scoring uncertainties. Second, all findings were based on the MESA dataset. Thus, additional investigation will be required in other populations. Third, cortical arousal and HRV response may not always be synchronous due to delayed reflection. Therefore, this study focused only on the cortical arousal events that overlap with deep learning model annotated arousal events for coupling analysis. Nevertheless, most of the cortical arousal events (n = 106,971) were included with the selected threshold. Moreover, the precision of the oximetry sensor may also affect the total counts of apneic and UOD arousals, as the apneic events were scored with a desaturation threshold of 3%. However, most of the UOD arousals (85%) in this study were not related to arousal etiologies after the secondary search. Lastly, in this study, a twenty-five second segment was employed to calculate ultra-short-term HRVs based on the analysis of the MESA dataset. A different window size may need to be selected for other populations.

Although this study has several limitations, our coupling analysis of arousal and HRVs has many strengths. To our knowledge, this is the first study to demonstrate HRV variations resulting from different cortical arousals using a large multi-center and multi-ethnic cohort. Arousal events can be accurately located using the deep learning model, leading to a better understanding of the instantaneous association between arousal and the cardiovascular system. Importantly, HRVs are associated with long-term cardiovascular disease (CVD) outcomes and altered HRVs may occur years earlier than the development and clinical recognition of CVD [37, 38]. The distinctiveness of HRVs between different genders and sleep stages found in this study may provide insights on association between arousal burden, long-term mortality and cardiac sudden death.

In conclusion, all arousals in this study were associated with an increased heart rate and HRVs. However, cardiovascular responses were also dependent on arousal causes, gender, and sleep stage during which arousal occurred. This study suggests that deep learning techniques can help analyze the instantaneous association between HRV and arousal in the general population. Future studies of arousal burden may take subpopulation and arousal types into account.

## Supporting information

S1 File Supplemental materials. More detailed information, such as adjusted p-values and effect sizes, was summarized in S1 File.
(PDF)
