# Supplementary material for: Coupling analysis of heart rate variability and cortical arousal using a deep learning algorithm
Source: PLoS One. 2023 Apr 6;18(4):e0284167. doi: 10.1371/journal.pone.0284167 (PMC10079022; doi:10.1371/journal.pone.0284167)
Supplement: S1 File — More detailed information, such as adjusted p-values and effect sizes, was summarized in S1 File. (PDF) [file pone.0284167.s001.pdf]

Supplementary information

Coupling analysis of heart rate variability and cortical arousal using a deep learning algorithm

Jiayan Huo M.S.[1], Stuart F. Quan M.D.[2,3], Janet Roveda Ph.D.[1,4,5], Ao Li Ph.D.[4,5]

1 Biomedical Engineering, The University of Arizona, Tucson, AZ, USA

2 Division of Sleep and Circadian Disorders, Departments of Medicine and Neurology, Brigham and Women's Hospital, Harvard Medical School, Boston, MA, USA

3 Asthma and Airway Disease Research Center, College of Medicine, The University of Arizona, Tucson, AZ, USA

4 Electrical and Computer Engineering, The University of Arizona, Tucson, AZ, USA

5 BIO5 Institute, The University of Arizona, Tucson, AZ, USA

Table S1. Comparison of demographic distributions of subjects before and after the subject exclusion

Characteristics	*Before exclusion (n=2237)*	*After exclusion (n = 1069)*
Female (%)	53.6%	53.5%
Age (mean ± SD)	69.0 ± 9.2	69.0 ± 8.9
Race		
Black, African American	27.5%	25.7%
Caucasian, White	37.1%	40.2%
Chinese American	11.8%	11.3%
Hispanic	23.5%	22.8%

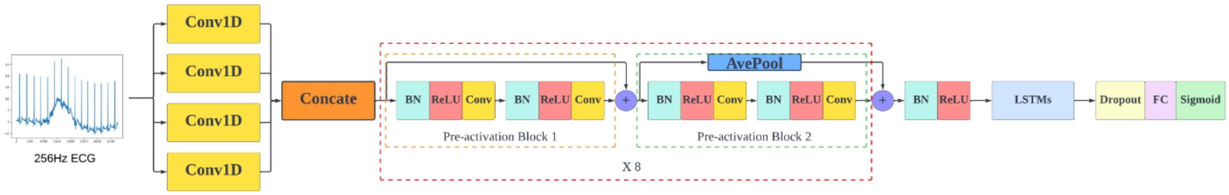

Figure S1. The full architecture of the deep learning model for arousal detection. Initially, the first layer consisted of 4 convolutions with different sizes of filters. Then the outputs of the first layer were concatenated and downsampled via two pre-activation residual blocks. The downsampling process was repeated eight times, followed by two stacked long-short term memory (LSTM) layers. Eventually, the signal was downsampled to 1 Hz, and a sigmoid function was employed to predict the arousal probability for each corresponding second. AvePool: Average Pooling; BN: Batch Normalization; Conv1D: 1d Convolution; FC: Fully Connected layer; LSTM: Long-short Term Memory; ReLU: Rectified of Linear Unit.

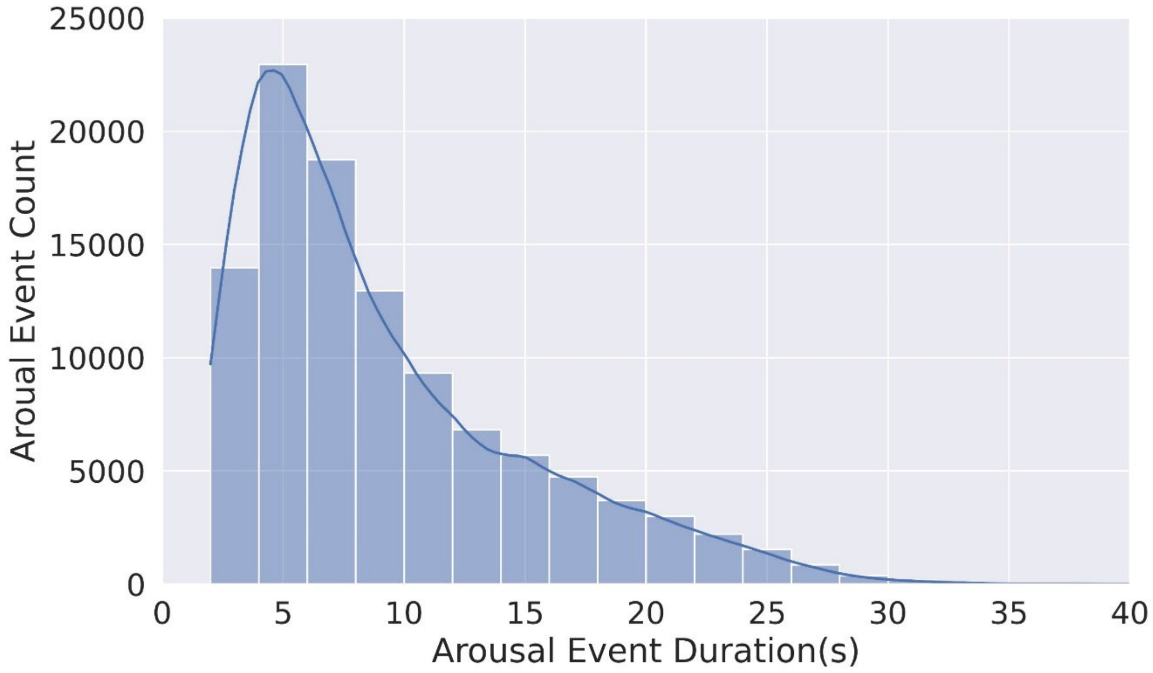

Figure S2. Arousal Event Durations

Table S2. Adjusted p-values and effect sizes of paired t-test on HRV parameters based on different types of arousal events between Pre-/Intra- and Intra-/Post-arousal segments

HRV Parameters	*segments*	*p-value (Effect size)*					
		OSA	CSA	Hypopnea	PLM	UOD	Spontaneous
SDNN	Pre vs Intra	0.0 (0.6)	3.0(0.2)	0.0 (0.6)	3.7e-108 (0.6)	0.0 (0.6)	0.0 (0.7)
	Intra vs Post	0.0 (0.7)	5.8e-10(0.4)	0.0 (0.5)	1.9e-111 (0.6)	0.0 (0.5)	0.0 (0.6)
RMSSD	Pre vs Intra	2.5e-6(0.1)	41.8(0.1)	1.6e-4(0.1)	0.5 (0.1)	4.7e-4 (0.1)	2.2e-8(0.1)
	Intra vs Post	4.4e-12(0.1)	9.9(0.12)	0.2 (0.0)	0.03 (0.1)	6.2e-4(0.1)	4.2e-13 (0.1)
pNN50	Pre vs Intra	3.6e-84(0.4)	8.8e-11(0.6)	2.4e-32(0.2)	3.1e-17(0.3)	3.1e-67(0.2)	3.6e-39(0.2)
	Intra vs Post	1.2e-98(0.4)	2.2e-8 (0.5)	1.3e-24(0.2)	3.7e-18(0.3)	8.1e-62(0.2)	5.3e-32(0.2)
HR	Pre vs Intra	0.0 (2.7)	1.9e-76(2.3)	0.0 (2.6)	3.9e-200 (1.9)	0.0 (2.5)	0.0 (2.2)
	Intra vs Post	0.0 (2.8)	1.0e-102 (3.0)	0.0 (2.6)	1.6e-234 (2.2)	0.0 (2.7)	0.0 (1.9)

CSA: Central Sleep Apnea; OSA: Obstructive Sleep Apnea; HR: Heart rate; PLM: Periodic Leg Movements; pNN50: Number of successive RR intervals pairs differing more than 50 ms divided by the total number of RR intervals; RMSSD: Square root of the mean squared differences between successive RR intervals; SDNN: Standard deviation of normal RR intervals; UOD: Undefined oxygen desaturation.

Table S3. Summary of HRV parameters and heart rate at different arousal stages between genders stratified by pathological arousals

HRVs	*Pre-arousal*		*Intra-arousal*		*Post-arousal*	
	Mean ± Standard Deviation					
OSA	Male	Female	Male	Female	Male	Female
SDNN [ms]	61.08 ± 54.50	48.77 ± 41.31	78.40 ± 48.33	68.40 ± 39.61	59.05 ± 51.81	48.51 ± 39.73
RMSSD [ms]	61.94 ± 87.58	43.03 ± 61.09	64.38 ± 76.18	48.94 ± 57.65	59.94 ± 84.82	43.18 ± 60.73
pNN50 [%]	17.02 ± 22.45	11.33 ± 16.84	20.21 ± 21.75	14.48 ± 16.14	16.44 ± 22.46	11.22 ± 16.85
HR [bpm]	61.50 ± 7.31	67.08 ± 8.91	66.22 ± 7.48	70.50 ± 8.81	61.84 ± 7.69	67.01 ± 8.88
CSA	Male	Female	Male	Female	Male	Female
SDNN [ms]	72.01 ± 67.09	60.61 ± 57.92	75.26 ± 61.34	67.11 ± 43.64	64.70 ± 62.45	52.17 ± 44.91
RMSSD [ms]	72.49±104.73	52.36±100.13	75.94±101.62	53.70 ± 71.54	70.41±101.94	49.28 ± 76.81
pNN50 [%]	14.94 ± 21.78	12.53 ± 19.13	18.23 ± 23.43	16.85 ± 20.00	14.87 ± 22.43	13.49 ± 18.87
HR [bmp]	63.59 ± 8.80	64.90 ± 9.07	66.46 ± 8.31	67.20 ± 8.80	63.34 ± 9.01	64.31 ± 8.05
Hypopnea	Male	Female	Male	Female	Male	Female
SDNN [ms]	49.42 ± 52.10	42.52 ± 41.61	61.85 ± 48.33	55.78 ± 41.29	52.36 ± 52.08	45.49 ± 42.01
RMSSD [ms]	54.17 ± 85.34	43.14 ± 63.80	54.99 ± 78.11	45.88 ± 62.39	54.60 ± 85.68	43.80 ± 63.95
pNN50 [%]	12.47 ± 20.09	11.41 ± 18.32	13.39 ± 18.98	12.48 ± 17.71	12.39 ± 19.79	11.65 ± 18.25
HR [bpm]	64.70 ± 8.10	66.88 ± 9.02	67.09 ± 8.19	68.95 ± 9.03	64.73 ± 8.15	66.66 ± 9.08
PLM	Male	Female	Male	Female	Male	Female
SDNN [ms]	56.55 ± 50.87	45.55 ± 40.36	66.65 ± 45.47	57.37 ± 39.66	57.57 ± 47.75	45.16 ± 38.80
RMSSD [ms]	56.32 ± 79.08	42.24 ± 60.78	57.17 ± 72.31	45.33 ± 58.50	56.72 ± 76.01	40.86 ± 59.78
pNN50 [%]	15.57 ± 21.07	11.18 ± 17.13	16.72 ± 20.59	12.80 ± 17.40	15.79 ± 20.97	10.87 ± 17.05
HR [bmp]	63.00 ± 7.63	65.85 ± 9.02	64.37 ± 7.63	66.90 ± 8.98	62.95 ± 7.74	65.73 ± 9.19
UOD	Male	Female	Male	Female	Male	Female
SDNN [ms]	58.06 ± 59.15	44.39 ± 41.02	71.21 ± 53.07	58.70 ± 40.35	58.64 ± 57.78	45.79 ± 40.79
RMSSD [ms]	63.85 ± 98.29	40.99 ± 59.72	64.28 ± 88.60	44.47 ± 57.96	63.24 ± 96.10	42.18 ± 61.29
pNN50 [%]	14.70 ± 21.87	11.58 ± 18.28	16.06 ± 20.93	13.18 ± 17.89	14.57 ± 21.47	11.77 ± 18.42
HR [bpm]	63.32 ± 7.88	67.01 ± 9.25	66.66 ± 8.13	69.55 ± 9.22	63.32 ± 8.01	66.80 ± 9.30
Spontaneous	Male	Female	Male	Female	Male	Female
SDNN [ms]	48.71 ± 52.06	39.55 ± 41.86	65.11 ± 49.02	56.85 ± 41.34	53.14 ± 51.52	44.45 ± 41.72
RMSSD [ms]	54.45 ± 83.53	42.84 ± 65.56	55.78 ± 76.40	45.90 ± 62.29	53.25 ± 80.26	42.79 ± 63.51
pNN50 [%]	13.62 ± 21.02	11.68 ± 19.15	14.56 ± 19.67	12.93 ± 17.96	13.60 ± 20.57	11.82 ± 18.68
HR [bpm]	63.55 ± 8.04	66.24 ± 8.64	65.94 ± 8.19	68.19 ± 8.80	63.72 ± 8.25	66.30 ± 8.94

bpm: Beats per minute; CSA: Central Sleep Apnea; OSA: Obstructive Sleep Apnea; HR: Heart rate; PLM: Periodic Leg Movements; pNN50: Number of successive RR intervals pairs differing more than 50 ms divided by the total number of RR intervals; RMSSD: Square root of the mean squared differences between successive RR intervals; SDNN: Standard deviation of normal RR intervals; UOD: Undefined oxygen desaturation.

Table S4. Adjusted p-values and effect sizes of t-test on HRV parameters means comparison among Pre-, Intra- and Post-arousal segments between different genders

HRV parameter	*segments*	*P-value (effect size)*					
		OSA	CSA	Hypopnea	PLM	UOD	Spontaneous
SDNN	Pre-	2.1e-28 (0.24)	12.7 (0.17)	8.3e-25 (0.15)	4.3e-16 (0.24)	3.4e-93 (0.26)	3.1e-46 (0.20)
	Intra-	8.4e-23 (0.22)	27.6 (0.14)	6.1e-21 (0.13)	5.3e-13 (0.22)	4.3e-92 (0.26)	1.9e-40 (0.18)
	Post-	8.0e-23 (0.22)	5.1 (0.21)	2.2e-24 (0.15)	5.4e-23 (0.29)	1.4e-85 (0.25)	3.8e-42 (0.19)
RMSSD	Pre-	6.5e-27 (0.24)	7.7 (0.20)	7.7e-25 (0.15)	5.0e-11 (0.20)	2.4e-100 (0.27)	2.8e-29 (0.16)
	Intra-	8.8e-23 (0.22)	2.9 (0.23)	4.4e-19 (0.13)	9.4e-9 (0.18)	8.0e-90 (0.26)	2.1e-24 (0.14)
	Post-	3.2e-22 (0.22)	4.3 (0.21)	1.3e-23 (0.14)	4.3e-15 (0.24)	2.5e-87 (0.25)	1.7e-25 (0.15)
pNN50	Pre-	4.4e-36 (0.28)	45.5 (0.11)	0.01 (0.05)	1.5e-14 (0.23)	1.5e-31 (0.15)	1.0e-10 (0.10)
	Intra-	3.8e-39 (0.29)	103.7 (0.06)	0.048 (0.05)	1.3e-11 (0.21)	6.6-29 (0.15)	1.76e-8 (0.09)
	Post-	3.0e-30 (0.25)	100.0 (0.06)	0.86 (0.04)	1.1e-18 (0.26)	8.8e-26 (0.14)	2.2e-9 (0.10)
HR	Pre-	1.6e-227 (0.7)	22.3 (0.15)	8.9e-75 (0.25)	1.7e-31 (0.34)	3.8e-254 (0.43)	3.4e-122 (0.32)
	Intra-	5.2e-135 (0.54)	68.8 (0.09)	5.8e-54 (0.22)	7.5e-25 (0.30)	3.2e-153 (0.34)	7.5e-83 (0.26)
	Post-	6.6e-187 (0.64)	48.3 (0.11)	6.4e-58 (0.22)	7.2e-29 (0.32)	1.2e-221 (0.41)	5.5e-106 (0.30)

CSA: Central Sleep Apnea; F: Female; M: Male; HR: Heart rate; OSA: Obstructive Sleep Apnea; PLM: Periodic Leg Movements; pNN50: Number of successive RR intervals pairs differing more than 50 ms divided by the total number of RR intervals; RMSSD: Square root of the mean squared differences between successive RR intervals; SDNN: Standard deviation of normal RR intervals; UOD: Undefined oxygen desaturation.

Table S5. Summary of HRVs and heart rate across different arousal stages between REM/NREM stratified by pathological arousals

HRVs	*Pre-arousal*		*Intra-arousal*		*Post-arousal*	
	Mean ± Standard Deviation					
OSA	REM	NREM	REM	NREM	REM	NREM
SDNN [ms]	49.46 ± 46.53	58.17 ± 51.22	75.09 ± 44.02	74.82 ± 46.01	47.99 ± 41.95	56.77 ± 49.17
RMSSD [ms]	48.79 ± 73.90	56.56 ± 80.83	50.96 ± 61.33	60.49 ± 72.16	46.11 ± 67.40	55.62 ± 79.44
pNN50 [%]	13.17 ± 19.92	15.37 ± 20.98	14.96 ± 17.40	18.80 ± 20.56	12.07 ± 19.17	15.09 ± 21.07
HR [bpm]	65.79 ± 9.23	63.03 ± 8.09	71.29 ± 9.21	67.05 ± 7.85	66.71 ± 9.38	63.08 ± 8.19
CSA	REM	NREM	REM	NREM	REM	NREM
SDNN [ms]	48.21 ± 42.93	71.51 ± 66.89	68.21 ± 43.66	74.30 ± 59.82	47.71 ± 37.70	63.69 ± 61.30
RMSSD [ms]	45.48 ± 73.52	70.79±105.98	53.80 ± 72.19	73.67 ± 99.33	49.02 ± 71.48	68.29±100.31
pNN50 [%]	11.54 ± 20.37	14.74 ± 21.47	15.17 ± 21.36	18.20 ± 23.06	14.10 ± 20.87	14.63 ± 21.95
HR [bmp]	66.66 ± 9.88	63.60 ± 8.76	69.64 ± 9.66	66.39 ± 8.27	66.63 ± 9.45	63.29 ± 8.79
Hypopnea	REM	NREM	REM	NREM	REM	NREM
SDNN [ms]	41.63 ± 45.48	46.44 ± 47.34	61.20 ± 42.84	58.46 ± 45.25	46.62 ± 46.78	49.16 ± 47.44
RMSSD [ms]	46.43 ± 73.96	48.80 ± 75.46	50.02 ± 67.43	50.40 ± 71.14	48.01 ± 74.93	49.25 ± 75.75
pNN50 [%]	10.21 ± 18.28	12.13 ± 19.32	12.91 ± 17.68	12.92 ± 18.44	11.01 ± 18.03	12.14 ± 19.15
HR [bpm]	66.19 ± 8.79	65.77 ± 8.63	69.14 ± 9.00	67.90 ± 8.62	65.73 ± 8.64	65.71 ± 8.70
PLM	REM	NREM	REM	NREM	REM	NREM
SDNN [ms]	45.23 ± 54.70	50.49 ± 45.35	63.24 ± 45.08	61.41 ± 42.51	45.04 ± 46.15	50.74 ± 43.33
RMSSD [ms]	49.20 ± 92.02	48.37 ± 69.11	52.19 ± 71.18	50.51 ± 65.07	46.52 ± 79.73	47.86 ± 67.56
pNN50 [%]	10.58 ± 20.85	13.16 ± 19.03	13.22 ± 19.33	14.55 ± 18.96	10.16 ± 19.96	13.09 ± 19.00
HR [bmp]	65.88 ± 8.92	64.59 ± 8.55	67.72 ± 9.00	65.76 ± 8.49	65.42 ± 9.07	64.50 ± 8.69
UOD	REM	NREM	REM	NREM	REM	NREM
SDNN [ms]	43.17 ± 46.16	53.71 ± 53.49	64.59 ± 42.24	66.09 ± 49.42	47.00 ± 46.51	54.18 ± 52.36
RMSSD [ms]	45.84 ± 74.53	55.48 ± 86.30	48.15 ± 61.88	57.12 ± 79.93	47.04 ± 73.62	55.47 ± 85.20
pNN50 [%]	11.06 ± 19.64	13.75 ± 20.59	13.10 ± 18.15	15.12 ± 19.99	11.43 ± 19.37	13.71 ± 20.41
HR [bpm]	65.96 ± 9.05	64.72 ± 8.61	70.14 ± 9.21	67.54 ± 8.60	66.10 ± 8.92	64.60 ± 8.71
Spontaneous	REM	NREM	REM	NREM	REM	NREM
SDNN [ms]	40.12 ± 43.77	43.86 ± 47.07	61.59 ± 41.56	60.32 ± 45.35	45.04 ± 44.36	48.56 ± 46.65
RMSSD [ms]	42.30 ± 67.25	48.42 ± 74.77	47.51 ± 62.21	50.45 ± 69.59	43.86 ± 67.89	47.70 ± 71.81
pNN50 [%]	10.57 ± 18.88	12.72 ± 20.11	13.19 ± 17.87	13.68 ± 18.83	11.36 ± 18.60	12.72 ± 19.64
HR [bpm]	65.66 ± 8.50	65.02 ± 8.48	67.91 ± 8.66	67.15 ± 8.61	64.75 ± 8.29	65.23 ± 8.78

bpm: Beats per minute; CSA: Central Sleep Apnea; HR: Heart rate; NREM: Non-rapid Eye Movement; OSA: Obstructive Sleep Apnea; PLM: Periodic Leg Movements; pNN50: Number of successive RR intervals pairs differing more than 50 ms divided by the total number of RR intervals; REM: Rapid Eye Movement; RMSSD: Square root of the mean squared differences between successive RR intervals; SDNN: Standard deviation of normal RR intervals; UOD: Undefined oxygen desaturation.

Table S6. Adjusted p-values and effect sizes of t-test on HRV parameters means comparison among Pre-, Intra- and Post-arousal segments between different sleep stages

HRV parameter	*segments*	*P-value (effect size)*					
		OSA	CSA	Hypopnea	PLM	UOD	Spontaneous
SDNN	Pre-	8.4e-8 (0.17)	2.7 (0.35)	5.0e-4 (0.10)	40.8 (0.12)	3.6e-26 (0.20)	0.14 (0.08)
	Intra-	165.9 (0.01)	94.1 (0.10)	1.0 (0.06)	127.5 (0.04)	17.25 (0.03)	46.4 (0.03)
	Post-	8.0e-9 (0.18)	12.8 (0.266)	2.7 (0.05)	29.8 (0.13)	3.3e-12 (0.14)	0.26 (0.08)
RMSSD	Pre-	0.05 (0.10)	18.1 (0.24)	29.3 (0.03)	179.2 (0.01)	6.7e-8 (0.11)	0.09 (0.08)
	Intra-	2e-4 (0.14)	31.4 (0.20)	160.7 (0.01)	155.6 (0.03)	3.7e-8 (0.12)	14.0 (0.04)
	Post-	0.002 (0.12)	34.7 (0.20)	89.6 (0.02)	165.6 (0.02)	5.3e-8 (0.10)	4.52 (0.05)
pNN50	Pre-	0.03 (0.11)	59.7 (0.15)	7.7e-4 (0.1)	27.5 (0.14)	8.9e-11 (0.13)	0.001 (0.11)
	Intra-	9e-10 (0.19)	71.4 (0.13)	195.0 (0.0)	87.7 (0.07)	3.4e-6 (0.10)	52.6 (0.03)
	Post-	2.8e-5 (0.15)	172.8 (0.02)	1.2 (0.06)	18.2 (0.15)	1.0e-7 (0.11)	0.63 (0.07)
HR	Pre-	4.3e-31 (0.33)	3.2 (0.35)	4.6 (0.05)	19.5 (0.15)	6.6e-13 (0.14)	0.3 (0.08)
	Intra-	5.3e-77 (0.53)	1.34 (0.39)	8.4 (0.14)	2.3 (0.23)	4.1e-59 (0.30)	0.03 (0.89)
	Post-	1.7e-52 (0.43)	1.7 (0.38)	182.7 (0.00)	49.1 (0.11)	6.3e-19 (0.17)	4.3 (0.05)

CSA: Central Sleep Apnea; HR: Heart rate; NREM: Non-rapid Eye Movement; OSA: Obstructive Sleep Apnea; PLM: Periodic Leg Movements; pNN50: Number of successive RR intervals pairs differing more than 50 ms divided by the total number of RR intervals; REM: Rapid Eye Movement; RMSSD: Square root of the mean squared differences between successive RR intervals; SDNN: Standard deviation of normal RR intervals; UOD: Undefined oxygen desaturation.

Comparison of HRV parameters associated with different types of arousals between OSA severity subpopulations

According to the apnea-hypopnea index (AHI), we stratified subjects into low-AHI (L) and high-AHI (H) using a cut-off value of 15. As shown in Figure S3, it is expected to see that the high-AHI group has more arousal events in total, especially arousal events induced by apnea, which was consistent with the AHI definition. In contrast, most of the arousals were spontaneous in the low-AHI subpopulation.

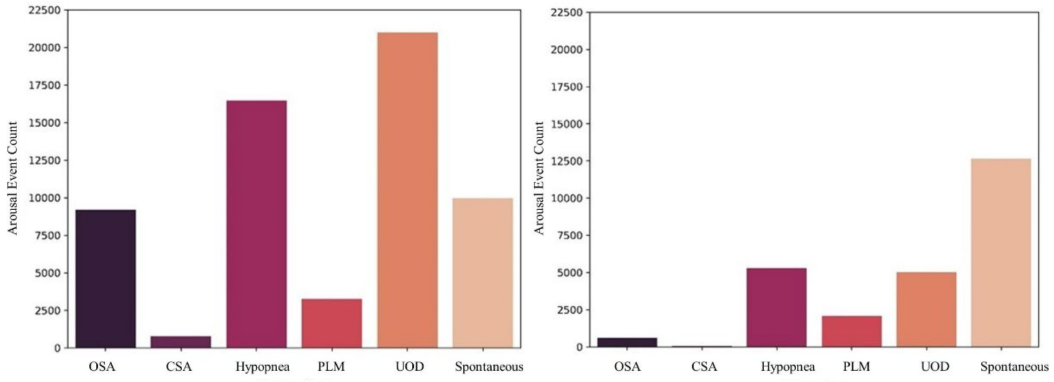

(a) High-AHI (b) Low-AHI

Figure S3. The distribution of arousal event in different OSA severity populations. (a) High-AHI (b) Low-AHI. AHI: Apnea-hypopnea Index; CSA: Central Sleep Apnea; OSA: Obstructive Sleep Apnea; PLM: Periodic Leg Movements; UOD: Undefined oxygen desaturation.

Table S7. Summary of HRVs and heart rate across different arousal stages between High- and Low-AHI groups stratified by pathological arousals

HRVs	*Pre-arousal*		*Intra-arousal*		*Post-arousal*	
	Mean ± Standard Deviation					
OSA	High-AHI	Low-AHI	High-AHI	Low-AHI	High-AHI	Low-AHI
SDNN [ms]	57.05 ± 50.85	52.72 ± 46.61	75.08 ± 45.85	72.69 ± 43.81	55.38 ± 48.44	55.25 ± 44.41
RMSSD [ms]	55.42 ± 80.12	54.20 ± 75.63	59.13 ± 70.65	57.30 ± 71.36	54.30 ± 78.41	51.17 ± 66.57
pNN50 [%]	15.19 ± 20.96	12.80 ± 18.70	18.44 ± 20.25	14.89 ± 18.14	14.71 ± 20.92	13.17 ± 18.92
HR [bpm]	63.36 ± 8.40	64.93 ± 7.46	67.62 ± 8.24	69.19 ± 7.99	63.55 ± 8.54	64.98 ± 7.70
CSA	High-AHI	Low-AHI	High-AHI	Low-AHI	High-AHI	Low-AHI
SDNN [ms]	70.58 ± 65.83	66.08 ± 66.22	74.47 ± 60.01	68.88 ± 46.45	63.30 ± 60.84	56.84 ± 52.60
RMSSD [ms]	69.62±102.61	66.31±120.63	73.57 ± 99.15	60.74 ± 81.86	67.63 ± 99.25	61.46 ± 92.57
pNN50 [%]	14.63 ± 21.30	13.87 ± 22.56	18.15 ± 23.01	16.68 ± 22.14	14.65 ± 21.88	14.85 ± 22.23
HR [bmp]	63.68 ± 8.90	64.94 ± 8.30	66.50 ± 8.47	67.30 ± 7.61	63.42 ± 8.96	64.19 ± 7.92
Hypopnea	High-AHI	Low-AHI	High-AHI	Low-AHI	High-AHI	Low-AHI
SDNN [ms]	46.44 ± 48.35	44.31 ± 43.35	58.13 ± 45.64	60.84 ± 42.94	49.03 ± 48.40	48.48 ± 44.03
RMSSD [ms]	49.03 ± 77.59	47.13 ± 67.85	50.60 ± 72.54	49.72 ± 64.77	49.61 ± 78.12	47.65 ± 67.40
pNN50 [%]	11.83 ± 19.14	12.23 ± 19.41	12.67 ± 18.32	13.77 ± 18.42	11.76 ± 18.84	12.82 ± 19.57
HR [bpm]	66.03 ± 8.61	65.11 ± 8.73	68.17 ± 8.59	67.59 ± 8.91	65.92 ± 8.64	65.04 ± 8.81
PLM	High-AHI	Low-AHI	High-AHI	Low-AHI	High-AHI	Low-AHI
SDNN [ms]	52.66 ± 48.35	46.74 ± 40.62	62.78 ± 43.86	59.33 ± 40.38	52.60 ± 45.64	47.41 ± 39.39
RMSSD [ms]	50.93 ± 75.26	44.44 ± 59.87	53.36 ± 70.62	46.16 ± 55.45	50.24 ± 73.38	44.02 ± 57.95
pNN50 [%]	13.45 ± 19.49	12.50 ± 18.36	15.01 ± 19.52	13.75 ± 18.06	13.31 ± 19.27	12.55 ± 18.64
HR [bmp]	64.30 ± 8.64	65.11 ± 8.41	65.59 ± 8.64	66.13 ± 8.28	64.24 ± 8.78	64.96 ± 8.56
UOD	High-AHI	Low-AHI	High-AHI	Low-AHI	High-AHI	Low-AHI
SDNN [ms]	54.10 ± 54.29	44.57 ± 44.32	67.04 ± 49.62	61.09 ± 43.00	54.26 ± 52.93	48.74 ± 45.66
RMSSD [ms]	56.47 ± 87.64	44.46 ± 71.03	57.87 ± 79.98	47.49 ± 66.51	56.05 ± 86.10	47.04 ± 72.58
pNN50 [%]	13.81 ± 20.71	11.61 ± 19.45	15.23 ± 20.00	13.23 ± 18.64	13.63 ± 20.44	12.42 ± 19.62
HR [bpm]	64.60 ± 8.55	66.09 ± 9.14	67.64 ± 8.58	68.91 ± 9.28	64.53 ± 8.60	65.96 ± 9.31
Spontaneous	High-AHI	Low-AHI	High-AHI	Low-AHI	High-AHI	Low-AHI
SDNN [ms]	47.61 ± 51.67	40.38 ± 42.41	63.11 ± 48.81	58.39 ± 41.77	51.81 ± 50.65	45.46 ± 42.66
RMSSD [ms]	53.26 ± 81.94	43.75 ± 67.26	55.54 ± 76.57	46.06 ± 62.12	52.77 ± 79.20	43.12 ± 64.46
pNN50 [%]	13.28 ± 20.43	11.95 ± 19.68	14.41 ± 19.37	13.05 ± 18.22	13.18 ± 19.78	12.15 ± 19.36
HR [bpm]	64.79 ± 8.51	65.30 ± 8.47	66.92 ± 8.50	67.45 ± 8.70	64.83 ± 8.66	65.46 ± 8.79

bpm: Beats per minute; CSA: Central Sleep Apnea; HR: Heart rate; OSA: Obstructive Sleep Apnea; PLM: Periodic Leg Movements; pNN50: Number of successive RR intervals pairs differing more than 50 ms divided by the total number of RR intervals; RMSSD: Square root of the mean squared differences between successive RR intervals; SDNN: Standard deviation of normal RR intervals; UOD: Undefined oxygen desaturation.

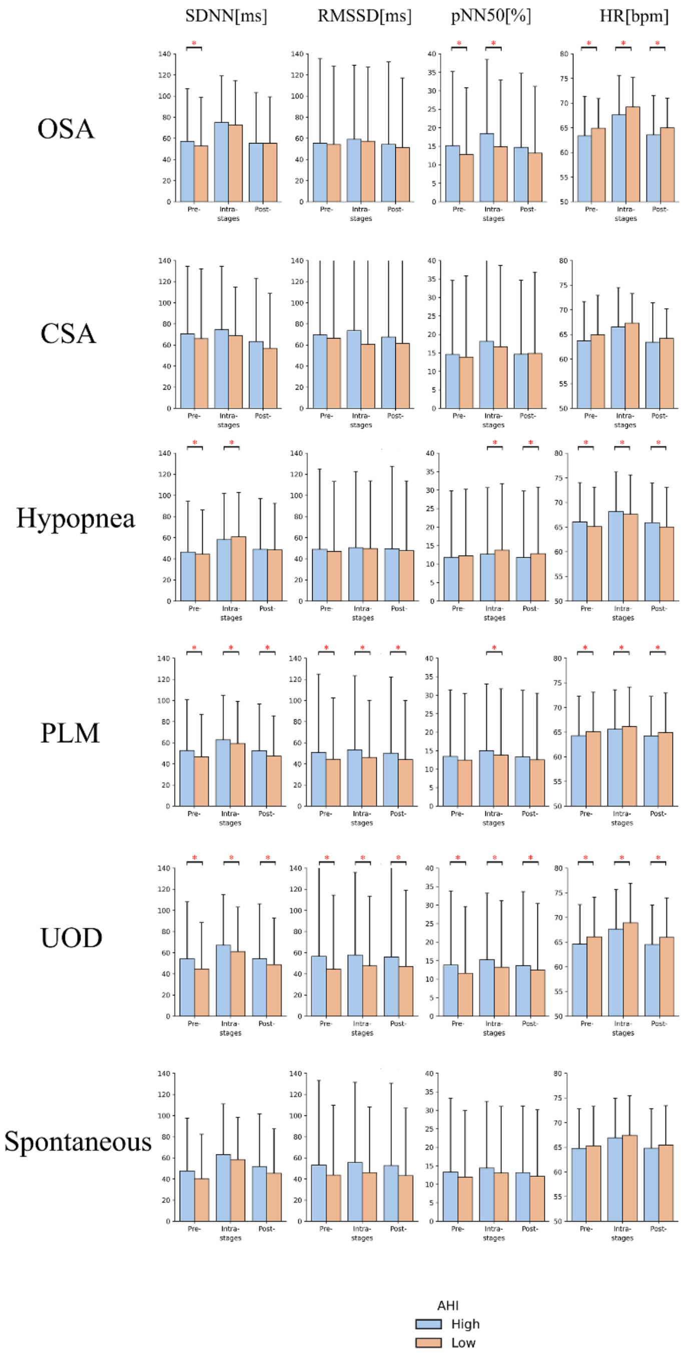

Figure S4. Barplot of parameters calculated from ECG signal across three defined segments of arousal events between High- and Low-AHI subpopulations. The asterisks indicate the pairwise statistical significance(adjusted p value < 0.05). CSA: Central Sleep Apnea; HR: Heart rate; NREM: Non-rapid Eye Movement; OSA: Obstructive Sleep Apnea; PLM: Periodic Leg Movements; pNN50: Number of successive RR intervals pairs differing more than 50 ms divided by the total number of RR intervals; REM: Rapid Eye Movement; RMSSD: Square root of the mean squared differences between successive RR intervals; SDNN: Standard deviation of normal RR intervals.

Table S8. Adjusted p-values and effect sizes of t-test on HRV parameters means comparison among Pre-, Intra- and Post-arousal segments between different AHI groups

HRV parameter	*segments*	*P-value (effect size)*					
		OSA	CSA	Hypopnea	PLM	UOD	Spontaneous
SDNN	Pre-	7.7 (0.09)	114.5 (0.07)	0.85 (0.05)	7.5e-4 (0.13)	1.7e-28 (0.18)	1.7e-28 (0.15)
	Intra-	41.5 (0.05)	86.6 (0.09)	0.03 (0.06)	0.8 (0.08)	1.0e-12 (0.12)	1.0e-12 (0.10)
	Post-	189.7 (0.00)	75.1 (0.11)	93.2 (0.01)	0.004 (0.12)	1.9e-9 (0.11)	2.5e-22 (0.14)
RMSSD	Pre-	142.6 (0.02)	158.8 (0.03)	22.3 (0.03)	0.19 (0.09)	3.6e-17 (0.14)	2.1e-19 (0.13)
	Intra-	106.1 (0.03)	55.7 (0.13)	85.4 (0.01)	0.017 (0.11)	3.4e-15 (0.13)	1.98e-22 (0.14)
	Post-	66.2 (0.04)	121.1 (0.06)	20.2 (0.03)	0.22 (0.09)	1.4e-9 (0.11)	1.2e-21 (0.14)
pNN50	Pre-	1.17 (0.11)	154.0 (0.04)	35.9 (0.02)	14.7 (0.05)	1.52e-9 (0.11)	1.5e-4 (0.07)
	Intra-	0.004 (0.18)	119.2 (0.06)	0.03 (0.06)	3.6 (0.07)	2.4e-8 (0.10)	1.3e-5 (0.07)
	Post-	14.4 (0.07)	187.7 (0.01)	0.08 (0.06)	30.9 (0.04)	0.03 (0.06)	0.02 (0.05)
HR	Pre-	0.001 (0.19)	48.0 (0.14)	3.9e-9 (0.11)	0.17 (0.09)	2.0e-25 (0.17)	9.7e-4 (0.06)
	Intra-	8.0e-4 (0.19)	86.2 (0.10)	0.005 (0.07)	4.8 (0.06)	3.3e-18 (0.15)	4.86e-4 (0.06)
	Post-	0.009 (0.17)	94.9 (0.09)	2.4e-8 (0.10)	0.7 (0.08)	2e-27 (0.16)	1.7e-5 (0.07)

CSA: Central Sleep Apnea; HR: Heart rate; OSA: Obstructive Sleep Apnea; PLM: Periodic Leg Movements; pNN50: Number of successive RR intervals pairs differing more than 50 ms divided by the total number of RR intervals; RMSSD: Square root of the mean squared differences between successive RR intervals; SDNN: Standard deviation of normal RR intervals; UOD: Undefined oxygen desaturation.

We also observed similar results in HRV variations in OSA, hypopnea, UOD and spontaneous arousals events compared to that of gender subpopulations (female vs low-AHI, male vs high-AHI). The result implies that gender (female) mainly contributed to the SDNN variation in CSA arousal, and SDNN variation in PLM arousal was related to OSA severity. All other variations in HRVs were affected by both gender and AHI. However, it should be noted that the correlation between gender and OSA severity may result in this similarity. The distribution of subjects indicated that those with the low-AHI were more likely to be female, as shown in Table S8.

Table S9 Subject distributions according to gender and AHI

AHI/Gender	*Female*	*Male*
AHI <= 15	307	164
AHI > 15	265	333

Though both gender and OSA severity may affect the HRVs response to arousal, the differences between Table S1 and Table S7 may provide insights on whether gender or AHI played a more important role in association between HRVs and arousal. Specifically, only female showed a significant reduction in SDNN after CSA arousal, implying the gender essentially affected SDNN of CSA arousal rather than AHI. We also found a significant decrease in SDNN after arousals resulted from PLM among the high-AHI subpopulation. Table S1 did not show any influence of PLM-induced arousal on HRV time-domain features among the male subpopulations, indicating OSA severity may affect the SDNN of PLM arousals.

Description and Reliability of Visual Arousal Scoring in MESA Polysomnograms

Polysomnograms in MESA were scored by certified technicians which included visual scoring of arousals. The sleep reading center for MESA performed interscorer reliability with absolute differences between scorers in the Arousal Index ranging from 1.93 to 7.23 /hour total sleep time (MESA Exam 5 Sleep Data Documentation Guide), Available at https://www.google.com/url?sa=t&rct=j&q=&esrc=s&source=web&cd=&ved=2ahUKEwjG7raeh8v8AhX7JUQIHfjkDPoQFnoECAsQAQ&url=https%3A%2F%2Fsleepdata.org%2Fdatasets%2Fmesa%2Ffiles%2Fdocumentation%2FMESA_Sleep_Data_Documentation_Guide.pdf&usg=AOvVaw3GRg5oa_Au9eZ7fT58.
